# Overexpression of STX11 alleviates pulmonary fibrosis by inhibiting fibroblast activation via the PI3K/AKT/mTOR pathway

**DOI:** 10.1038/s41392-024-02011-y

**Published:** 2024-11-11

**Authors:** Guichuan Huang, Xiangsheng Yang, Qingyang Yu, Qun Luo, Chunrong Ju, Bangyan Zhang, Yijing Chen, Zihan Liang, Shu Xia, Xiaohua Wang, Dong Xiang, Nanshan Zhong, Xiao Xiao Tang

**Affiliations:** 1 State Key Laboratory of Respiratory Disease, National Clinical Research Center for Respiratory Disease, National Center for Respiratory Medicine, Guangzhou Institute of Respiratory Health, The First Affiliated Hospital of Guangzhou Medical University, Guangzhou, China; 2 Guangzhou Laboratory, Bio-island, Guangzhou, China

**Keywords:** Pathogenesis, Diseases

## Abstract

Fibroblast activation plays an important role in the occurrence and development of idiopathic pulmonary fibrosis (IPF), which is a progressive, incurable, and fibrotic lung disease. However, the underlying mechanism of fibroblast activation in IPF remains elusive. Here, we showed that the expression levels of STX11 and SNAP25 were downregulated in the lung tissues from patients with IPF and mice with bleomycin (BLM)-induced lung fibrosis as well as in the activated fibroblasts. Upregulation of STX11 or SNAP25 suppressed TGF-β1-induced activation of human lung fibroblasts (HLFs) via promoting autophagy. However, they failed to suppress fibroblast actviation when autophagy was blocked with the use of chloroquine (CQ). In addition, STX11 or SNAP25 could inhibit TGF-β1-induced fibroblast proliferation and migration. In vivo, overexpression of STX11 exerted its protective role in the mice with BLM-induced lung fibrosis. STX11 and SNAP25 mutually promoted expression of each other. Co-IP assay indicated that STX11 has an interaction with SNAP25. Mechanistically, STX11-SNAP25 interaction activated fibroblast autophagy and further inhibited fibroblast activation via blocking the PI3K/AKT/mTOR pathway. Overall, the results suggested that STX11-SNAP25 interaction significantly inhibited lung fibrosis by promoting fibroblast autophagy and suppressing fibroblast activation via blocking the PI3K/ATK/mTOR signaling pathway. Therefore, STX11 serves as a promising therapeutic target in IPF.

## Introduction

Idiopathic pulmonary fibrosis (IPF), a chronic, progressive and fatal interstitial lung disease with unclear etiology, is characterized by repetitive alveolar epithelial cell injuries, fibroblast proliferation and differentiation into myofibroblast (namely activated fibroblast) as well as overwhelming extracellular matrix accumulation in the lung interstitium.^1,2^ Activated fibroblasts are the key effector cells in the development of IPF. They exhibit strong contractile ability as well as produce and release a large amount of collagen proteins with subsequent destruction and abnormal repairment of lung tissues.^1,2^ The median survival time of patients with IPF after diagnosis is 2–3 years and the 5-year survival rate is below 40%.^3^ Despite extensive research efforts, no effective drugs are available up to now. Two drugs, nintedanib (a tyrosine kinase inhibitor) and pirfenidone, have been developed and approved for the treatment of patients with IPF. They shed light on decelerating lung function decline, but failed to halt or reverse disease progression and reduce the mortality of IPF patients.^1^ Hence, it is imperative to unravel the pathogenesis and develop molecular targeted drugs for IPF.

Soluble N-ethylmaleimide-sensitive factor attachment protein receptor (SNARE) proteins are common fusogenic factors that contribute to intracellular trafficking of cargo and membrane fusion.^4^ SNARE complexes consist of bundles of coiled-coil with four helices.^5^ The Qa-, Qb-, and Qc-SNARE patterns belong to SNAREs from the syntaxin (STX) and synaptosome associated protein 25 (SNAP-25) families (including SNAP23, SNAP25, SNAP29 and SNAP47), whereas the R-SNARE pattern is presented in the vesicle-associated membrane protein (VAMP).^5^ STX11, a SNARE family member, is highly expressed in immune system, including spleen, thymus, and lymph nodes. It is widely expressed in various organs as well, with a relative high expression in lung and low expression in heart, liver, and kidney tissues.^6^ STX11 is closely related to cellular biological activities as well as the occurrence and development of diseases. For instance, Kinoshita et al.^7^ reported that it regulated the stimulus-dependent transport of TLR4 to the plasma membrane by cooperating with SNAP-23 in macrophages, which is associated with microbial pathogenesis and immune responses. STX11 mutation causes familial hemophagocytic lymphohistiocytosis type 4, which is a life-threatening disease characterized by severe hyperinflammation.^8^ Kögl et al. found that after infecting with lymphocytic choriomeningitis virus, Stx11-deficient mice displayed severely reduced degranulation and cytolytic activity of cytotoxic T lymphocytes and natural killer cells and exhibited all clinical symptoms of hemophagocytic lymphohistiocytosis.^8^ We found that STX11 is downregulated in IPF lung based on the data mining in GEO public database. However, its role in IPF remains unexplored.

Autophagy (a term originated from Greek for “self-eating”), a critical cellular process recycling cytosolic long-lived proteins and organelles to retain cellular homeostasis,^9^ is associated with a wide variety of physiological and pathological processes.^10^ Under physiological conditions, a constitutive basal level of autophagy is required to maintain cell homeostasis. For instance, basal level of autophagy slows cellular senescence via cleaning impaired mitochondria to decrease reactive oxygen species production and maintain the physiological activities of cells. Whereas abnormal autophagy results in a series of diseases, including asthma,^11^ chronic obstructive pulmonary disease^11^ and IPF.^12^ For instance, Zhang et al.^13^ reported that lncIAPF (lncRNA inhibit autophagy in pulmonary fibrogenesis), aslo termed as LINC00941, promoted fibroblast-to-myofibroblast differentiation as well as myofibroblast proliferation and migration via suppressing the fusion between autophagosome and lysosome during autophagy, accelerating the progression of pulmonary fibrosis. Another study showed that interleukin-37 (IL-37) was downregulated in IPF lung tissues and overexpression of IL-37 alleviated bleomycin (BLM)-induced lung fibrosis in mice by inhibiting the transforming growth factor-β1 (TGF-β1) signaling pathway and enhancing autophagy in IPF fibroblasts.^14^ Therefore, autophagy is cloesly related to the progression of pulmonary fibrosis. The endosome-lysosome fusion is a crucial step during autophagy process. Lysosome associated membrane protein 1 (LAMP1) is a marker of late endosomes/lysosomes. Offenhauser and colleagues found that GFP-STX11 was predominantly located in LAMP1-labeled late endosomes and concluded that STX11 regulates the fusion between endosome and lysosome via binding to Vti1b in macrophages.^15^ However, whether STX11 affects the occurrence and progression of IPF by regulating autophagy remains unknown.

In the current study, we examined the expression of STX11 and SNAP25 in the lung tissues from IPF patients and mice with BLM-induced pulmonary fibrosis as well as in the activated fibroblasts. Then, we further explored the role and underlying mechanism of STX11 and SNAP25 in fibroblast-to-myofibroblast differentiation. We also investigated whether overexpression of STX11 alleviated BLM-induced pulmonary fibrosis in mice. In addition, we further identified the role of STX11 in human lung fibroblast (HLF) activaiton with the use of recombinant human STX11 protein and found that it also inhibited TGF-β1-induced expression of α-SMA, fibronectin, and collagen I, indicating that STX11 inhibited TGF-β1-induced HLF activation. Our study aimed to clarify the contribution of STX11 to fibroblast activaion and its underlying mechanism as well as to identify novel potential therapeutic targets for the treatment of IPF.

## Results

### The expression of STX11 is decreased in lung tissues from patients with IPF and mice with BLM-induced pulmonary fibrosis

In order to explore the expression of STX11 in lung fibrosis, we screened the database related to IPF in GEO database. As shown in Supplementary Fig. 1a–f, the expression of STX11 was significantly decreased in IPF group compared with the control group (GSE10667, GSE24206, GSE32537, GSE53845, GSE72073, GSE110147). Even though no statistical significance was observed in GSE21369 and GSE35145 data sets, its expression in IPF group exhibited a decreasing trend (Supplementary Fig. 1g, h). Additionally, we performed a meta-analysis of STX11 expression data from GEO database. As shown in Supplementary Fig. 1i, STX11 expression was markedly lower in IPF group (SMD = −1.88, 95% CI: −2.59 to −1.18, *p* < 0.0001).

**Fig. 1:**
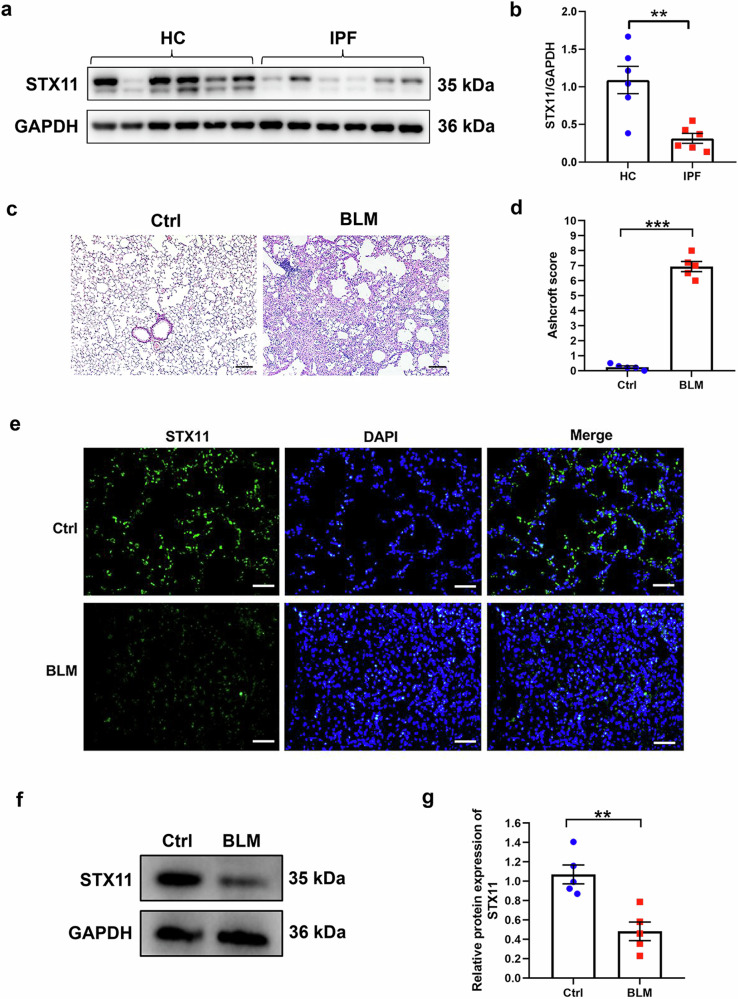
The expression of STX11 in IPF and control lung tissues from the clinical specimen and mice. **a**, **b** The protein expression of STX11 in human IPF tissues and in normal human lung tissues was detected by western blot assay. **c** H&E staining was conducted on mice lung tissue sections from BLM group and control group. **d** The Ashcroft score was used to evaluate the degree of fibrosis in mice lung tissues. Immunofluorescence (**e**) and western blot (**f**, **g**) assays were applied to detect the expression of STX11 in mice lung tissues (original magnification ×200, scale bar: 100 μm). Data were expressed as mean ± SEM (*n* = 5 or 6). *p < 0.05; **p < 0.01; ***p < 0.001

We further verified STX11 expression in clinical specimen and mice lung tissues. The western blot showed reduced STX11 expression in human IPF lung tissues as compared to health control (HC) lung tissues (Fig. 1a, b). Meanwhile, we intratracheally instilled BLM to induce lung fibrosis in mice. H&E results showed damaged alveolar structure, accumulated collagen, thickened interlobular septa, increased Ashcroft score in BLM group, indicating that BLM indeed induced lung fibrosis in mice (Fig. 1c, d). We then examined its expression in mice lung tissues. Decreased expression of STX11 was observed in BLM group (Fig. 1e–g). Altogether, these results demonstrated reduced expression of STX11 in lung fibrosis, suggesting its possible involvement in the progression of lung fibrosis.

### STX11 expression is decreased in TGF-β1-induced fibroblast activation

Fibroblast activation plays a key role in the pathogenesis of IPF. In order to establish a cell model of fibroblast activation, we treated human lung fibroblasts (HLFs) with TGF-β1. The qPCR and western blot results showed enhanced expression of ACTA2 (gene encoding α-SMA), fibronectin (gene encoding fibronectin), and COL1A1 (gene encoding collagen I) in TGF-β1 group as compared with control group (Supplementary Fig. 2a–e). These data confirmed activation of HLFs by TGF-β1.

**Fig. 2:**
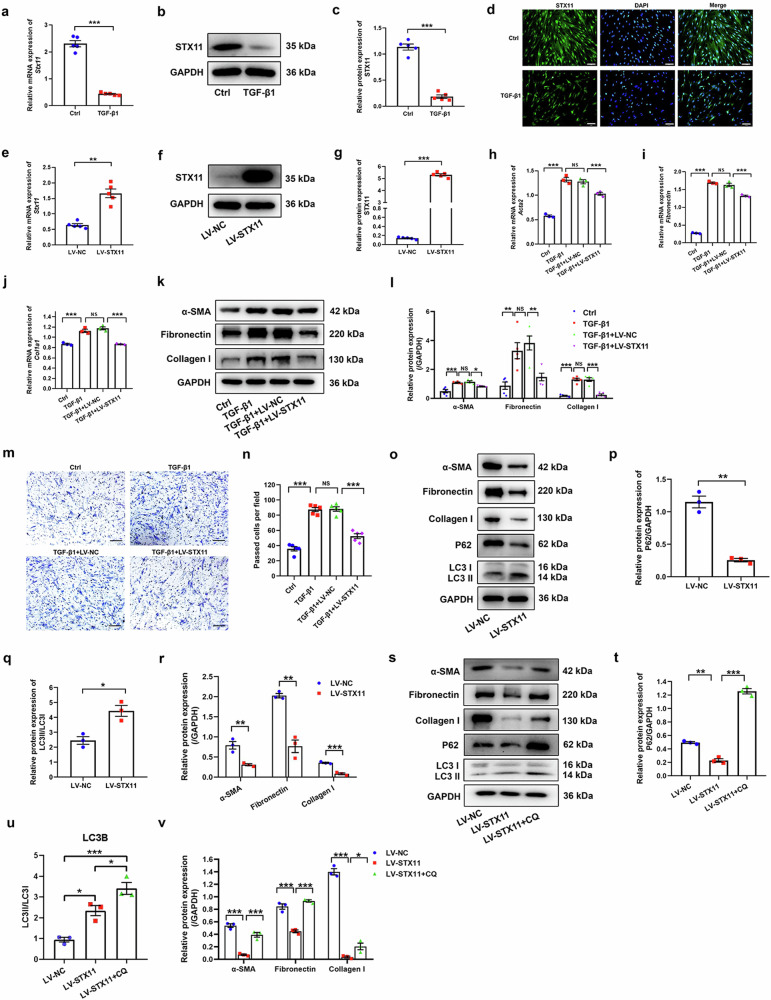
The role of STX11 in fibroblast autophagy and activation. HLFs were treated with 10 ng/ml TGF-β1 for 48 h. **a** qPCR assay was used to detect the mRNA expression of STX11. **b**, **c** Western blot assay was used to detect the protein expression of STX11. **d** Immunofluorescence assay was used to detect the protein expression of STX11 (original magnification ×200, scale bar: 100 μm). HLFs were infected with lentiviruses harboring STX11 and treated with TGF-β1 for 48 h. qPCR (**e**) and western blot (**f**, **g**) assays were used to detect the expression of STX11. HLFs with STX11 overexpression were treated with 10 ng/ml TGF-β1 for 48 h. The expression of α-SMA, fibronectin, and COL1A1 (collagen I) was examined by qPCR (**h**–**j**) and western blot (**k**, **l**) assays. **m**, **n** Transwell assay was used to determine the migration ability of HLFs (original magnification ×100, scale bar: 200 μm). **o**–**r** Western blot assay was used to determine the expression of fibroblast activation markers (α-SMA, fibronectin, collagen I) and autophagy-associated markers (LC3II/I, P62) in HLFs with STX11 overexpression. **s**–**v** HLFs with STX11 overexpression were stimulated with chloroquine (CQ) at a final concentration of 10 mM for 48 h. Western blot assay was used to examine the expression of fibroblast activation markers and autophagy-associated markers in HLFs. GAPDH was used as an internal control. Data were expressed as mean ± SEM (*n* = 3 or 5). *p < 0.05; **p < 0.01; ***p < 0.001; NS, no significance

We then identified STX11 expression in HLFs treated with TGF-β1. The mRNA and protein expression of STX11 was significantly decreased in TGF-β1 group (Fig. 2a–d), suggesting that HLF activation downregulated STX11 expression and STX11 might be involved in fibroblast activation.

### Overexpression of STX11 attenuates fibroblast activation via promoting autophagy

To reveal what role STX11 plays in fibroblast activation, we stably overexpressed STX11 in HLFs with lentivirus (LV). The mRNA and protein expression levels of STX11 were markedly upregulated in LV-STX11 group compared with LV-NC group (Fig. 2e–g). Forced expression of STX11 dramatically decreased TGF-β1-induced expression of α-SMA, fibronectin, and collagen I (Fig. 2h–l). In addition, we further identified the role of STX11 in HLF activaiton with the use of recombinant human STX11 protein and found that it also inhibited TGF-β1-induced expression of these fibroblast activation markers, indicating that STX11 inhibited TGF-β1-induced HLF activation (Supplementary Fig. 3). Meanwhile, we investigated the role of STX11 in migration of HLFs. As illustrated in Fig. 2m, n, TGF-β1 promoted migration of HLFs. And STX11 inhibited TGF-β1-induced migration of HLFs. We also explored the role of STX11 in HLF proliferation. Ki-67 protein has been widely utilized as a cell prolifertaion marker for decades. Therefore, to reflect the proliferation of HLFs, we decteded Ki-67 expression using immunofluorescence assay. pcDNA-STX11 plasmids were employed to overexpress STX11 in HLFs. As shown in Supplementary Fig. 4, TGF-β1 promoted proliferation of HLFs; whereas STX11 suppressed TGF-β1-induced HLF proliferation. In order to explore whether STX11 inhibited HLF activation via promoting autophagy, we examined the autophagy-related markers (LC3II/I and p62) and fibroblast activation markers (α-SMA, fibronectin, and collagen I) of HLFs in the absence or presence of an autophagy inhibitor (chloroquine, CQ). As shown in Fig. 2o–r, overexpression of STX11 resulted in decreased protein expression of α-SMA, fibronectin, collagen I, and p62 as well as increased ratio of LC3II/LC3I. To further determine the role of STX11 in autophagy, we transfected tandem-tagged mCherry-GFP-LC3 adenovirus into HLFs to monitor the subcellular localization of LC3. As shown in Supplementary Fig. 5, autophagosomes were increased in pcDNA-STX11 group, indicating that STX11 overexpression promoted autophagy of HLFs.

CQ can increase the pH value of acidic lysosomes and inactivate acidic hydrolases in lysosomes, and then inhibit the fusion and degradation of autophagic lysosomes within cells. Therefore, cells treated with CQ lead to the accumulation of LC3 II/LC3 I and p62, indicating blockage of autophagy. As expected, CQ treatment increased the protein expression of p62 and LC3 II/LC3 I, indicating that CQ can inhibit autophagy in HLFs (Fig. 2s–u). In addition, CQ treatment promoted the protein expression of fibroblast activation markers (Fig. 2s–v). These findings demonstrated that STX11 inhibited fibroblast-to-myofibroblast differentiation via activating autophagy.

### STX11 interacts with SNAP25

**Fig. 3:**
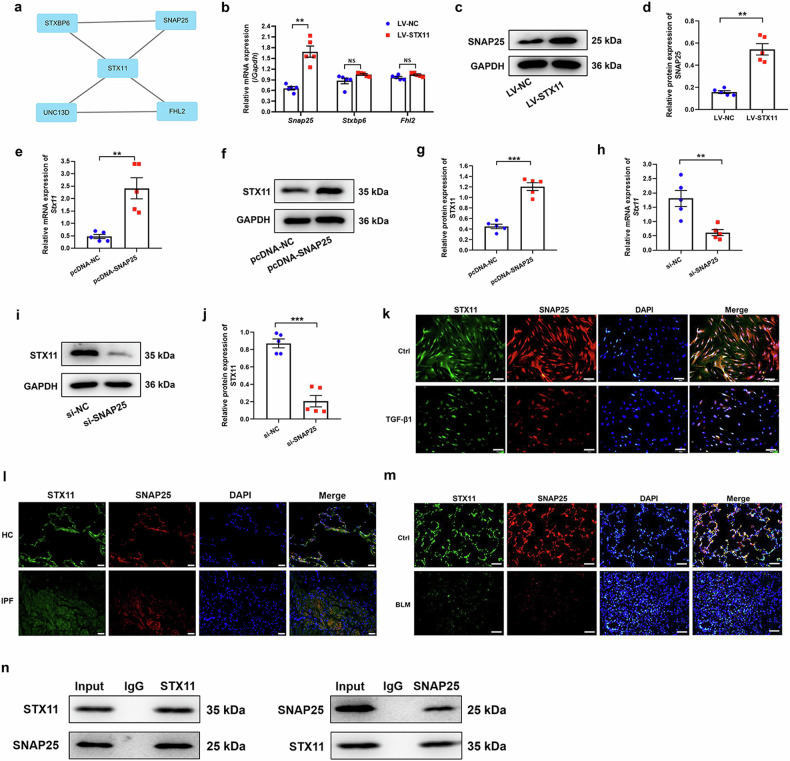
The relationship between STX11 and SNAP25. **a** The STRING online tool was used to identify the interaction protein with STX11. qPCR (**b**) and western blot (**c**, **d**) assays were used to detect the expression of SNAP25. qPCR (**e**) and western blot (**f**, **g**) assays were utilized to examine the expression of STX11 in HLFs transfected with pcDNA-NC or pcDNA-SNAP25. qPCR (**h**) and western blot (**i**, **j**) assays were conducted to explore the expression of STX11 in HLFs transfected with si-NC or si-SNAP25. **k** Immunofluorescence assay was performed to detect the expression of STX11 and SNAP25 in HLFs (original magnification ×200, scale bar: 100 μm). **l** Representative images showing STX11 (green), SNAP25 (red) and DAPI (blue) in HC and IPF lung sections by immunofluorescence staining (scale bar = 100 μm). **m** Immunofluorescence assay was performed to detect the expression of STX11 and SNAP25 in mice lung tissues (original magnification ×200, scale bar: 100 μm). **n** Reciprocal immunoprecipitation of STX11 and SNAP25 in HLFs. GAPDH was used as an internal control. Data were expressed as mean ± SEM (*n* = 5). **p < 0.01; ***p < 0.001

We employed STRING database to explore the molecules that interact with STX11 and the analysis indicated that STX11 might have a relationship with SNAP25, STXBP6, UNC13D, and FHL2 (Fig. 3a). We detected the mRNA expression of these genes in STX11-overexpressed HLFs. As shown in Fig. 3b–d, STX11 promoted SNAP25 expression at both mRNA and protein levels. Next, we determined STX11 expression in HLFs with SNAP25 knockdown or overexpression. Interestingly, we found that upregulation of SNAP25 also promoted STX11 expression (Fig. 3e–g); conversely, downregulation of SNAP25 inhibited STX11 expression (Fig. 3h–j).

We further investigated whether STX11 interacted with SNAP25. First, we simultaneously detected the expression of STX11 and SNAP25 in the mouse lung tissue and HLFs by immunofluorescence. We found co-localized fluorescence between STX11 and SNAP25 in the human lung tissue, mouse lung tissue and HLFs (Fig. 3k–m), indicating a potential interaction between STX11 and SNAP25. Then, we conducted co-IP assay to validate whether STX11 could bind to SNAP25. The results showed that STX11 protein or SNAP25 protein could pull down each other, suggesting that STX11 and SNAP25 had an interaction and formed a complex (Fig. 3n).

### Overexpression of SNAP25 attenuates fibroblast activation and proliferation

**Fig. 4:**
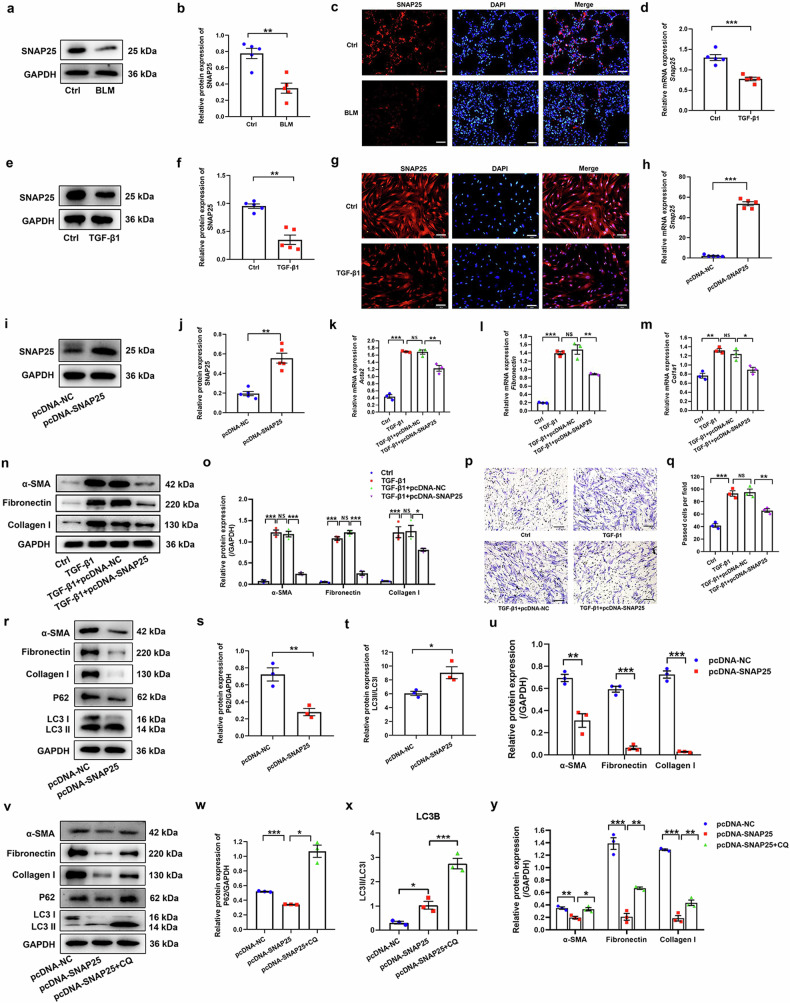
The role of SNAP25 in fibroblast autophagy and activation. **a**, **b** Western blot assay was used to detect the protein expression of SNAP25 in the mice lung. **c** Immunofluorescence assay was used to detect the protein expression of SNAP25 in the mice lung (original magnification ×200, scale bar: 100 μm). HLFs were treated with 10 ng/ml TGF-β1 for 48 h. qPCR (**d**) and western blot (**e**, **f**) assays were used to detect the expression of SNAP25 in HLFs. **g** Immunofluorescence assay was used to detect the protein expression of SNAP25 (original magnification ×200, scale bar: 100 μm). HLFs were transfected with pcDNA-NC or pcDNA-SNAP25 for 48 h. qPCR (**h**) and western blot (**i**, **j**) assays were conducted to identify the expression of SNAP25. HLFs were transfected with pcDNA-NC or pcDNA-SNAP25 for 24 h, and then stimulated with TGF-β1 for 48 h. qPCR (**k**–**m**) and western blot (**n**, **o**) assays were performed to determine the expression of α-SMA, fibronectin, and collagen I. **p**, **q** Transwell assay was used to determine the migration ability of HLFs (original magnification ×100, scale bar: 200 μm). **r**–**u** Western blot assay was used to determine the expression of fibroblast activation markers (α-SMA, fibronectin, collagen I) and autophagy-associated markers (LC3II/I, P62) in HLFs with enhanced expression of SNAP25. **v**–**y** HLFs with SNAP25 overexpression were stimulated with chloroquine (CQ) at a final concentration of 10 mM for 48 h. Western blot assay was used to detect the expression of fibroblast activation markers and autophagy-associated markers in HLFs. GAPDH was used as an internal control. Data were expressed as mean ± SEM (*n* = 3 or 5). *p < 0.05; **p < 0.01 ; ***p < 0.001

We detected SNAP25 expression in the lung tissues of mice with BLM-induced lung fibrosis and TGF-β1-activated fibroblasts. Decreased expression of SNAP25 was found in BLM group (Fig. 4a–c). Likewise, its expression in TGF-β1-activated group was lower than that in the control group (Fig. 4d–g). These results demonstrated that SNAP25 might be invloved in lung fibrosis and fibroblast activation.

To assess the role of SNAP25 in fibroblast activation, we transfected pcDNA-SNAP25 or SNAP25 siRNA into HLFs. PcDNA-SNAP25 remarkably promoted SNAP25 expression in HLFs (Fig. 4h-j). As displayed in Fig. 4k–o, the expression of fibroblast activation markers was significantly reduced in SNAP25-overexpressed HLFs. Meanwhile, SNAP25 overexpression inhibited migration of HLFs (Fig. 4p, q). We also explored the role of SNAP25 in HLF proliferation. As shown in Supplementary Fig. 6, TGF-β1 promoted proliferation of HLFs; whereas SNAP25 overexpression inhibited TGF-β1-induced HLF proliferation, demonstrating that overexpression of SNAP25 impeded fibroblast activation, proliferation and migration.

In contrast, downregulation of SNAP25 by siRNA (Supplementary Fig. 7a–c) increased expression of markers for fibroblast activation as well as promoted HLF migration (Supplementary Fig. 7d–j). These results demonstrated that SNAP25 silencing promoted fibroblast activation and migration.

To explore whether SNAP25 inhibited HLF activation via promoting autophagy, we detected autophagy-related markers and fibroblast activation markers of HLFs in the absence or presence of CQ. As shown in Fig. 4r–u, overexpression of SNAP25 resulted in decreased protein expression of α-SMA, fibronectin, collagen I, and p62 and increased LC3II/LC3I. To further determine the role of SNAP25 in autophagy, we transfected tandem-tagged mCherry-GFP-LC3 adenovirus into HLFs to monitor the subcellular localization of LC3. As shown in Supplementary Fig. 8, increased autophagosomes were seen in pcDNA-SNAP25 group, indicating that SNAP25 overexpression promoted autophagy of HLFs.

CQ treatment increased the protein expression of p62 and LC3 II/LC3 I, indicating the blockage of autophagy in HLFs (Fig. 4v–x). In addition, the protein expression of fibroblast activation markers was increased post CQ treatment (Fig. 4v–y). These findings demonstrated that SNAP25 inhibited fibroblast-to-myofibroblast differentiation via activating autophagy.

### Forced expression of STX11 attenuates fibroblast activation via upregulating SNAP25

**Fig. 5:**
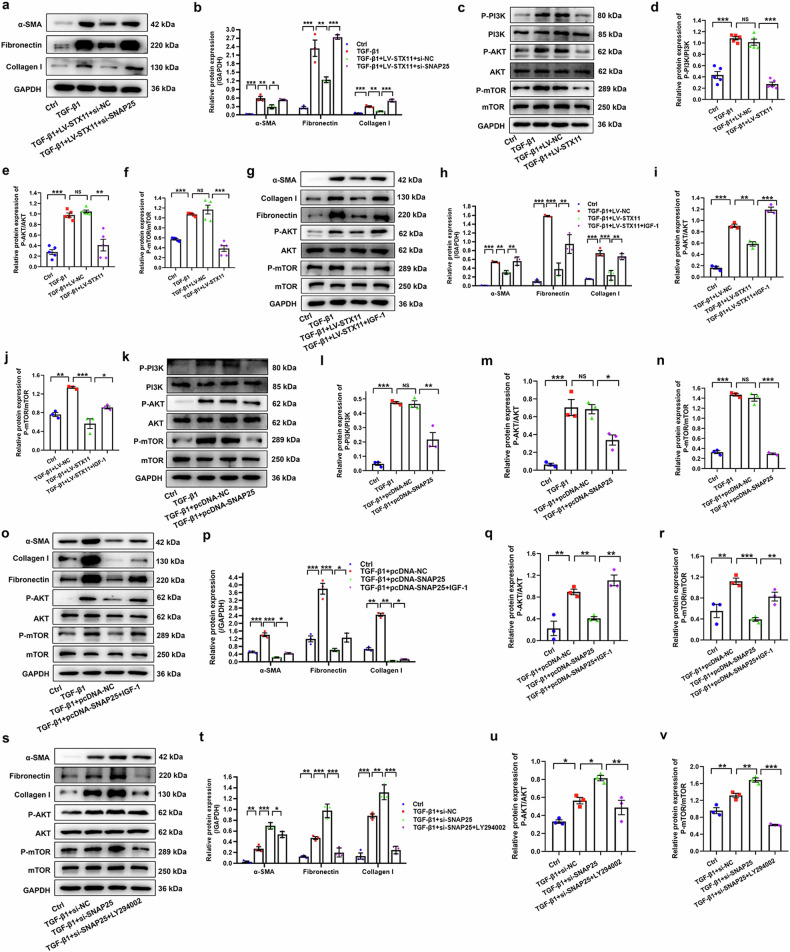
The effect of STX11 and SNAP25 on the PI3K/AKT/mTOR pathway. **a**, **b** HLFs with STX11 overexpression were transfected with si-NC or si-SNAP25 for 24 h, and then stimulated with TGF-β1 for 48 h. Western blot assay was performed to determine the expression of α-SMA, fibronectin, and collagen I. **c**–**f** The expression of the PI3K/AKT/mTOR pathway related proteins was detected by western blot in HLFs with STX11 overexpression. **g**–**j** HLFs with forced expression of STX11 were pretreated with 100 ng/ml IGF-1 for 1 h, and exposed to TGF-β1 for 48 h. Western blot assay was utilized to detect the protein expression in HLFs. **k**–**n** The expression of the PI3K/AKT/mTOR pathway related proteins was detected by western blot in HLFs with SNAP25 overexpression. **o**–**r** HLFs with forced expression of SNAP25 were pretreated with 100 ng/ml IGF-1 for 1 h, and exposed to TGF-β1 for 48 h. Western blot assay was utilized to detect the protein expression in HLFs. **s**–**v** HLFs with SNAP25 silencing were pretreated with 10 μM LY294002 for 1 h, and exposed to TGF-β1 for 48 h. Western blot assay was utilized to detect the protein expression in HLFs. Data were expressed as mean ± SEM (*n* = 3 or 5). *p < 0.05; **p < 0.01 ; ***p < 0.001; NS, no significance

To determine whether STX11 inhibited fibroblast activation via upregulating SNAP25, we treated HLFs with lentivirus-STX11 and SNAP25 siRNA. As expected, upregulation of STX11 inhibited the expression of fibroblast activation markers, whereas this process could be reversed by downregulation of SNAP25, suggesting that STX11 inhibited fibroblast activation by upregulating SNAP25 (Fig. 5a, b).

### STX11-SNAP25 complex attenuates fibroblast activation via blocking the PI3K/AKT/mTOR signaling pathway

To illustrate the mechanism underlying suppressed activation of HLFs by STX11-SNAP25 complex, we explored the relevant signaling pathways. The PI3K/AKT/mTOR pathway plays a vital part in autophagy.^16^ Hence, we explored whether it was involved in the inhibition of fibroblast activation by STX11 and SNAP25. Western blot showed that this pathway was activated by TGF-β1, whereas suppressed by the enhanced expression of STX11 (Fig. 5c–f). In order to further determine whether STX11 exerts its effect on fibroblast activation in a PI3K/AKT/mTOR signaling pathway-dependent way, we treated HLFs with IGF-1, a PI3K/AKT/mTOR activator. The alterations in fibroblast activation markers and relevant proteins of the PI3K/AKT/mTOR pathway caused by STX11 overexpression could be reversed by IGF-1 (Fig. 5g–j). Collectively, these results suggested that STX11 inhibited fibroblast activation by suppressing this pathway.

We also examined the relationship between SNAP25 and the PI3K/AKT/mTOR pathway. As expected, forced expression of SNAP25 inhibited this pathway (Fig. 5k–n). However, the decreased expression of phospho-AKT, phospho-mTOR, a-SMA, fibronectin, and collagen I by forced expression of SNAP25 was elevated again after treating with IGF-1 (Fig. 5o–r). In contrast, downregulation of SNAP25 activated the pathway, which was reversed by the pathway inhibitor LY294002 (Fig. 5s–v). Taken together, these data indicated that SNAP25 suppressed fibroblast activation by blocking the PI3K/AKT/mTOR signaling pathway.

### Enhanced expression of STX11 attenuates BLM-induced pulmonary fibrosis in mice

**Fig. 6:**
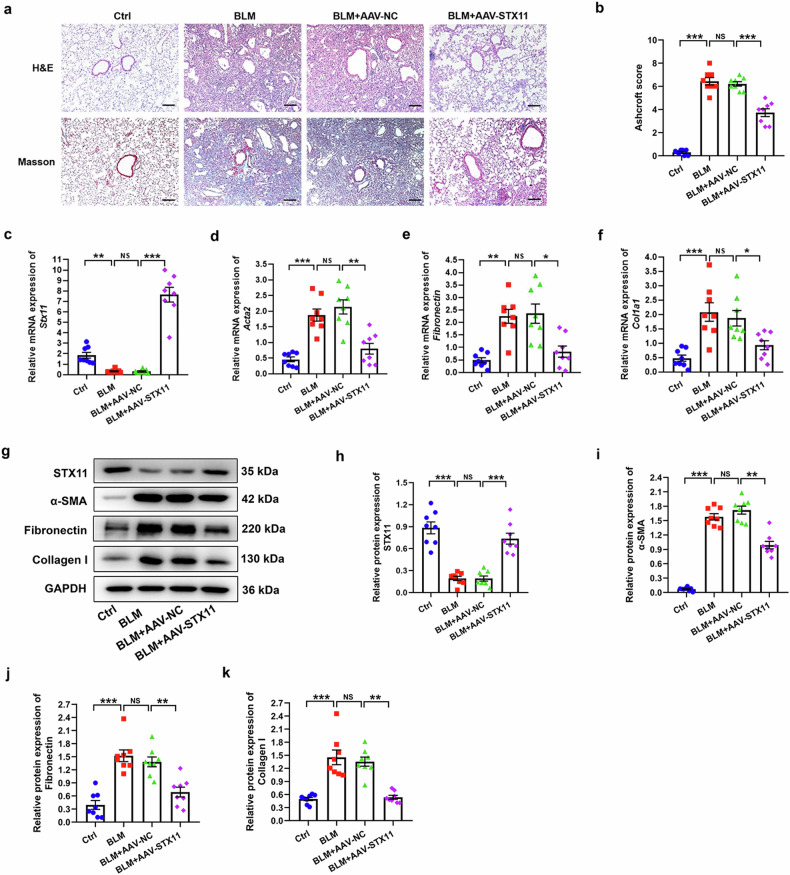
Overexpression of STX11 inhibits BLM-induced pulmonary fibrosis in mice. **a** Representative images of H&E and Masson staining in the lung sections of mice. **b** Ashcroft score based on H&E staining was determined. **c**–**f** The mRNA expression of STX11, α-SMA, fibronectin, and COL1A1 was detected by qPCR. **g**–**k** The protein expression of STX11, α-SMA, fibronectin, and collagen I was detected by western blot. GAPDH was used as an internal control. Data were expressed as mean ± SEM (*n* = 8). *p < 0.05; **p < 0.01; *** *p* < 0.001; NS, no significance

To explore the role of STX11 in BLM-induced lung fibrosis, we intratracheally instilled AAV-STX11 into the mice lung. H&E and Masson staining revealed that lung fibrosis developed in the BLM group (Fig. 6a, b). After AAV-STX11 instillation, STX11 expression was obviously elevated, which accompanied with decrease of lung fibrosis (Fig. 6a–c, g–h). Moreover, the expression of those fibroblast activation-related markers was increased in BLM and BLM + AAV-NC group as compared with Ctrl group (Fig. 6d–f, g, i–k). Whereas, the above-mentioned markers were reduced in BLM + AAV-STX11 group compared with BLM + AAV-NC group (Fig. 6d–f, g, i–k). These results indicated that overexpression of STX11 alleviated BLM-induced lung fibrosis in mice.

## Discussion

In the current study, we investigated the role of STX11 in IPF. The Gene Expression Omnibus (GEO) database is a public repository that archives and freely distributes gene expression data sets of various diseases, including lung cancer and IPF. First, we found that STX11 was markedly downregulated in the lung tissues from IPF patients (GEO database and clinical samples) as well as mice with BLM-induced lung fibrosis. We found that STX11 was also decreased in the activated lung fibroblasts. Then, overexpression of STX11 inhibited HLF activation via promoting HLF autophagy and reduced the severity of BLM-induced pulmonary fibrosis in mice. In addition, we also clarified the effect of STX11 on activaiton of human lung fibroblasts by using recombinant human STX11 protein and found that it inhibited TGF-β1-induced expression of fibroblast activation markers as well. Meanwhile, gain function of SNAP25 inhibited HLF activation via promoting autophagy. In contrast, loss of SNAP25 promoted HLF activation. Thorough STRING database and co-IP assay, we identified that STX11 could form a complex with SNAP25, blocking the PI3K/AKT/mTOR pathway. Our study indicated STX11 as a potential therapeutic target for IPF.

The abnormal expression of STX11 is related to familial hemophagocytic lymphohistiocytosis type 4.^17^ Cui et al.^18^ reported STX11 as a key gene in IPF but they found no statistical significance of its mRNA expression in the lung tissues between normal group and IPF group. We inferred that the small number (*n* = 3) of clinical samples in their study may be the reason.

It is well known that fibroblasts are major effector cells in lung fibrosis. Fibroblast activation results in a large amount of extracellular matrix deposition, leading to lung fibrosis.^19^ An escalating number of studies indicated that autophagy is essential in fibroblast activation.^20^ As TGF-β1 is an important cytokine that induces fibroblast activation, we used it to activate HLFs. We found that TGF-β1 decreased STX11 expression in HLFs, indicating that STX11 may be involved in HLF activation. A previous study showed that STX11 attenuates adipose-triglyceride-lipase action by regulating lipolysis and lipophagy in hepatocytes.^21^ Offenhauser and colleagues demonstrated that STX11 is predominantly located in LAMP1-labeled late endosomes and hence concluded that STX11 regulates the fusion between endosome and lysosome via binding to Vti1b in macrophages.^15^ And as we know, the endosome-lysosome fusion is an important step during autophagy. STX11, a member of SNARE family, is related to autophagy.^4^ We found that STX11 overexpression promoted autophagy and inhibited activation of HLFs; in addition, HLFs were activated again after blocking the autophagy with CQ (an inhibitor of autophagy). These findings indicated that forced expression of STX11 inhibited HLF activation via promoting autophagy. Furthermore, overexpression of STX11 suppressed HLF activation with the increase of SNAP25.

As a SNARE complex, SNAP25 is vital in regulating synaptic messaging, neurotransmitter release, vesicle extravasation, and intercellular signaling.^22,23^ It is also associated with vesicle fusion and lysosomal trafficking.^24^ Studies have shown dysregulated expression of SNAP25 in various cancers. For example, SNAP25 was upregulated in lung cancer,^25^ whereas downregulated in prostate cancer.^26^ In our study, overexpression of STX11 enhanced SNAP25 expression, suggesting that SNAP25 might also participate in HLF activation. To address this question, we detected SNAP25 expression in the lung tissues of BLM-induced mice and TGF-β1-activated HLFs. Decreased SNAP25 expression was found in both experiments. Furthermore, enhanced SNAP25 expression inhibited fibroblast activation, while downregulation of it promoted fibroblast activation. Studies have shown that SNAP25 was related to autophagy as well. For instance, Mu et al. reported that nuclear protein transcription regulator 1 (NUPR1) mediated autolysosomal efflux via promoting SNAP25 expression in tumor cells.^22^ Another study found that SNAP25 was deficient in Niemann-Pick type C disease-specific induced neural stem cells (NPC-iNSCs) and SNAP25 overexpression in NPC-iNSCs significantly decreased expression of p62 and LC3-I/II, indicating that upregulation of SNAP25 could enhance autophagy.^27^ In addition, Wang et al. found that SNAP25 boosted PTEN-induced kinase 1 (PINK1)-dependent mitophagy and inhibited caspase-3/GSDME-dependent pyroptosis to exert neuroprotective effects against postoperative cognitive dysfunction.^28^ Here, we found that overexpression of SNAP25 promoted autophagy and inhibited activation of HLFs.

STX, VAMP and SNAP-25 subfamilies belong to the SNARE family.^5^ The SNARE complex is crucial in calcium-dependent exocytosis of synaptic vesicles and intracellular trafficking of cargo and promotes membrane fusion.^4^ A study showed that STX1A and SNAP25 interacted with each other and formed a tight complex in vitro to exert biological roles.^29^ Nozawa et al. reported that STX6-VTI1B-VAMP3 complex promoted xenophagy via mediating the fusion between autophagosomes and recycling endosomes.^30^ Interestingly, here we revealed that STX11 and SNAP25 boosted expression of each other. In addition, co-IP assay showed that STX11 could interact with SNAP25, indicating that they formed a complex to exert the biological roles.

The PI3K/AKT/mTOR pathway mediates cell proliferation, differentiation, survival and is tightly linked to lung fibrosis.^31,32^ It is well-established that PI3K is composed of a p85 subunit and a p110 subunit. Once PI3K is activated, its p85 subunit translocates to the plasma membrane, and P110 subunit catalyzes PIP2 substrate to PIP3 via interacting with p85 subunit. PIP3 then interacts with AKT, phosphorylating Akt protein at Thr308 and Ser473 sites. Finally, AKT activates downstream target molecules, including mTOR.^33,34^ Evidences suggested that this pathway participated in pulmonary fibrosis via inhibiting autophagy of lung fibroblasts.^35^ For example, Peng et al. found that aspirin alleviated lung fibrosis via a PI3K/AKT/mTOR-dependent autophagy pathway.^35^ Li et al. reported that duvelisib alleviated pulmonary fibrosis via enhancing autophagy and suppressing activation of lung fibroblasts via blocking the PI3K/AKT/mTOR pathway.^34^ In our study, we found that STX11 and SNAP25 complex promotes fibroblast autophagy, and thereby inhibits fibroblast activation by suppressing this signaling pathway.

There are restrictions in this study. First, the mechanism underlying mutual regulation of STX11 and SNAP25 is not yet clear. Second, the specific binding site between STX11 and SNAP25 requires to be identified in further study.

**Fig. 7:**
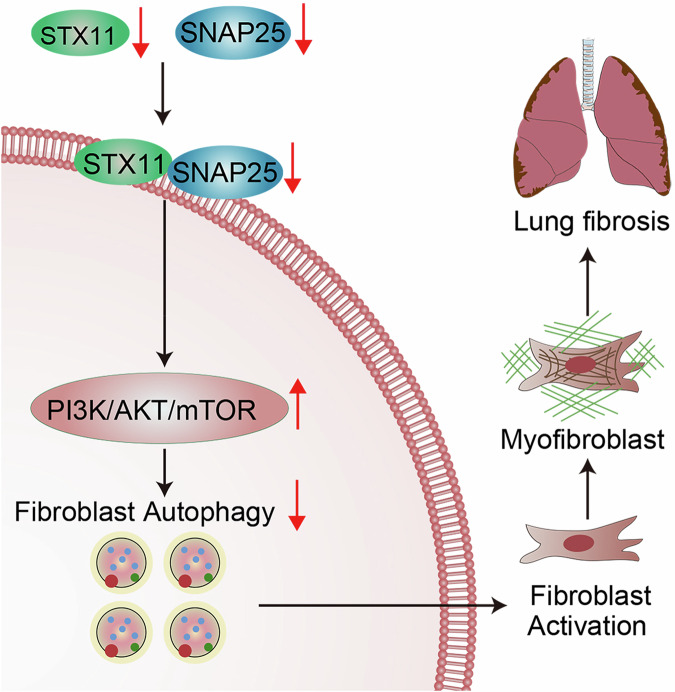
Schematic diagram of the study. STX11 forms a complex with SNAP25, blocking the PI3K/AKT/mTOR pathway and promoting autophagy of lung fibroblasts, which inhibits the development of lung fibrosis

In conclusion, we found that STX11-SNAP25 interaction inhibited fibroblast activation via blocking the PI3K/AKT/mTOR signaling pathway (Fig. 7). These results not only broaden our understanding of IPF pathogenesis and the mechanism governing fibroblast activation, and may also provide insights for the development of novel anti-IPF molecular-targeted drugs.

## Materials and Methods

### Data collection from Gene Expression Omnibus (GEO) database

The microarray data used in this study were obtained from the GEO database (https://www.ncbi.nlm.nih.gov/). The keywords were utilized as follows: (pulmonary fibrosis OR lung fibrosis). The results were further filtered as follows: Series [Entry type], Homo sapiens [Organism], and Expression profiling by array [Study type]. There were two inclusion criteria for microarray dataset in the study. First, the dataset included non-fibrotic and fibrotic human lung tissues. Second, the dataset offered the expression value of STX11 in non-fibrotic and fibrotic human lung tissues.

### Human lung tissues

The study protocol involving human participants was approved by the Ethics Committee of The First Affiliated Hospital of Guangzhou Medical University (approval no. 2018-92) and all patients provided written informed consent. IPF patients were included in the study according to the diagnostic criteria of 2018 ATS/ERS/JRS/ALAT Clinical Practice Guideline.^36^ Tissue from explanted IPF lungs or healthy donors was collected for validation of STX11 expression.

### Animal experiments

Healthy C57BL/6 mice (8 weeks old) were purchased from Hua Fu Kang Co. Ltd. (Beijing, China). Mice were kept on a regular diet in a specific pathogen-free conditions with an environmental temperature control at 21–24 °C and a 12 h light-dark cycle. The mice received intraperitoneal pentobarbital sodium and were intratracheally instillated 50 μl BLM as previously described.^37^ Mice in the control group received the same volume of saline.

To assess the role of STX11 in the mice lung, we divided the mice into following groups: (1) saline control; (2) BLM; (3) BLM + AAV-NC; (4) BLM + AAV-STX11. AAV-NC or AAV-STX11 was instilled into the lung of mice three weeks before BLM instillation. Three weeks post BLM instillation, all mice were sacrificed and the mice lung tissues were harvested. All animal procedures were approved by the Ethics Committee of The First Affiliated Hospital of Guangzhou Medical University (No. 2022559).

### Cell culture

Human lung fibroblasts (HLFs) were purchased from ATCC. Cells were cultured in DMEM medium containing 10% FBS, 100 U/mL penicillin and 100 μg/mL streptomycin at 37 °C in a humidified incubator with 5% CO_2_. HLFs were activated by incubating with 10 ng/ml transformation growth factor-β1 (TGF-β1).

### Transfection

HLFs (1.5 × 10^5^) were seeded in 6-well plates overnight and then were transfected with pcDNA-NC, pcDNA-SNAP25, siRNA-NC or siRNA-SNAP25 using transfection reagent (Polyplus, France) following the manufacturer’s protocol.

### Quantantive real-time PCR (qPCR)

Total RNA was extracted from the mice lung or HLFs using NucleoZOL reagent and reverse transcribed to cDNA using the Hifair® III 1st Strand cDNA Synthesis SuperMix. cDNA was further amplified by SYBR Green Master Mix (Yeasen, Shanghai, China). Relative gene expression was normalized to GAPDH and calculated using the 2^△△^Ct method. The primers were listed in Table 1.

**Table 1:** The sequence of primers in this study

Gene	Species	Forward (3’−5’)	Reverse (3’−5’)
STX11	Human	GATGAAGCAGCGCGACAAC	AAAACACGTCCCACTTACCCT
SNAP25	Human	TCGTGTAGTGGACGAACGG	TCTCATTGCCCATATCCAGGG
Fibronectin	Human	CGGTGGCTGTCAGTCAAAG	AAACCTCGGCTTCCTCCATAA
COL1A1	Human	GAGGGCCAAGACGAAGACATC	CAGATCACGTCATCGCACAAC
ACTA2	Human	GCTGGTGATGATGCTCCCA	GCCCATTCCAACCATTACTCC
GAPDH	Human	CTTTGGTA TCGTGGAAGGACTC	GTAGAGGCAGG GATGATGTTCT
Stx11	mouse	AGATGAAACAGCGCGACAACTG	GCTCGAACATGTCCTCAATCTGC
Fibronectin	mouse	GTGTAGCACAACTTCCAATTACGAA	GGAATTTCCGCCTCGAGTCT
Col1a1	mouse	CTGGCGGTTCAGGTCCAAT	TTCCAGGCAATCCACGAGC
Acta2	mouse	GCTGGTGATGATGCTCCCA	GCCCATTCCAACCATTACTCC
Gapdh	mouse	GACATCAAGAAGGTGGTGAAGC	GAAGGTGGAAGAGTGGGAGTT

### Western blot

Total protein was extracted from HLFs or the lung of IPF patients or mice using RIPA lysis buffer, supplemented with protease/phosphatase inhibitor cocktail. Protein concentration was quantified by the BCA protein assay kit (Yeasen, Shanghai, China). Proteins (20 to 30 μg) were separated using SDS-PAGE and transferred to PVDF membranes (Millopore, USA). After blocking with 5% non-fat milk for 1 h at room temperature, the membranes were incubated with primary antibodies at 4 °C overnight and were washed three times with TBST and incubated with secondary antibodies for 1 h at room temperature. Finally, proteins were visualized using TIAN LONG Imaging system. The antibody information was as follows: anti-STX11 (1:1000, 13301-1-AP, Proteintech), anti-SNAP25 (1:1000, 14903-1-AP, Proteintech), anti-α-SMA (1:1000, ab5694, Abcam), anti-Fibronectin (1:1000, sc-8422, Santa cruz), anti-Collagen I (1:1000, 14695-1-AP, Proteintech), anti-AKT (1:1000, Proteintech), anti-phospho-AKT (Ser473) (1:1000, Proteintech), anti-PI3K (1:1000, Abcam), anti-phospho-PI3K (Tyr607) (1:1000, Abcam), anti-mTOR (1:1000, ab32028, Abcam), anti-phospho-mTOR (phospho S2448) (1:1000, ab109268, Abcam).

### Transwell assay

Migration of HLFs was assessed by transwell assay. A total of 8 × 10^3^ to 1 × 10^4^ HLFs were seeded in the upper chamber of transwell plates with an 8.0 μm pore size. HLFs were transfected with pcDNA-SNAP25 or siRNA-SNAP25 for 24 h, and then treated with TGF-β1 for 48 h. Three visual fields of each group were randomly selected for cell counting.

### Immunofluorescence

HLFs were seeded on coverslips at 1 × 10^4^ cells per well per 24-well plate. After TGF-β1 stimulation, cells were rinsed with PBS three times and fixed with 4% paraformaldehyde for twenty minutes at room temperature. Then, cells were permeabilized in 0.2% Triton-100 buffer. After blocking in 5% goat serum albumin for 1 h at room temperature, cells were incubated with the following primary antibodies at 4 °C overnight: anti-STX11(1:100, 13301-1-AP, Proteintech), anti-SNAP25 (1:100, sc-20038, Santa cruz). anti-Ki-67 (1:100, ab15580, Abcam), anti-α-SMA (1:100, 67735-1-lg, Proteintech). Cells were then rinsed with PBS three times and were incubated with goat anti-rabbit IgG-H&L (FITC) (1:500, ab6717, Abcam) or goat anti-mouse IgG H&L (Alexa Fluor®594) (1:500, ab150116, Abcam) for 1 h at room temperature.

### Co-immunoprecipitation (Co-IP)

HLFs were lysed in IP lysis buffer (Abclonal, RM00022, China) supplemented with protease/phosphatase inhibitor cocktail. Cell lysates were then incubated with protein A/G PLUS-Agarose (Santa cruz, sc-2003, USA) at 4 °C for 1 h. The supernatant was incubated with primary antibodies for IP and protein A/G PLUS-Agarose at 4 °C overnight. The mixture was centrifuged at 3500 rpm for 10 min and the sendiment was collected and mixed with loading buffer for western blot assay.

### Statistical analysis

Data were expressed as mean ± standard error of the mean (SEM) and were analyzed with IBM SPSS software (Version 26.0). Student’s unpaired t test was used for comparisons between two groups. One-way analysis of variance with Tambane’s T2 (equal variances not assumed) or LSD post hoc test (equal variances assumed) was utilized to analyze datasets containing multiple groups. *p* < 0.05 was regarded as statistically significant. *p < 0.05; **p < 0.01; ***p < 0.001; NS, no significant difference.

## Supplementary information

Supplementary Materials

## Data Availability

All the data generated or analyzed in this study are included in this published article and supplementary files.
