# Supplementary material for: Overexpression of STX11 alleviates pulmonary fibrosis by inhibiting fibroblast activation via the PI3K/AKT/mTOR pathway
Source: Signal Transduct Target Ther. 2024 Nov 11;9:306. doi: 10.1038/s41392-024-02011-y (PMC11551190; doi:10.1038/s41392-024-02011-y)
Supplement: Supplementary file 1 — Supplementary Materials [file 41392_2024_2011_MOESM1_ESM.docx]

Supplementary Materials for

**Overexpression of STX11 alleviates pulmonary fibrosis by inhibiting fibroblast activation via the PI3K/AKT/mTOR pathway**

Guichuan Huang^1^, Xiangsheng Yang^1^, Qingyang Yu^1^, Qun Luo^1^, Chunrong Ju^1^, Bangyan Zhang^1^, Yijing Chen^1^, Zihan Liang^1^, Shu Xia^1^, Xiaohua Wang^1^, [Dong Xiang](https://pubmed.ncbi.nlm.nih.gov/?sort=date&term=Xiang+D&cauthor_id=38053854)^1^, Nanshan Zhong^1,2^*, Xiao Xiao Tang^1,2^*

Correspondence to: tangxiaoxiao@gird.cn

nanshan@vip.163.com

**This PDF file includes:**

Figures. S1 to S8


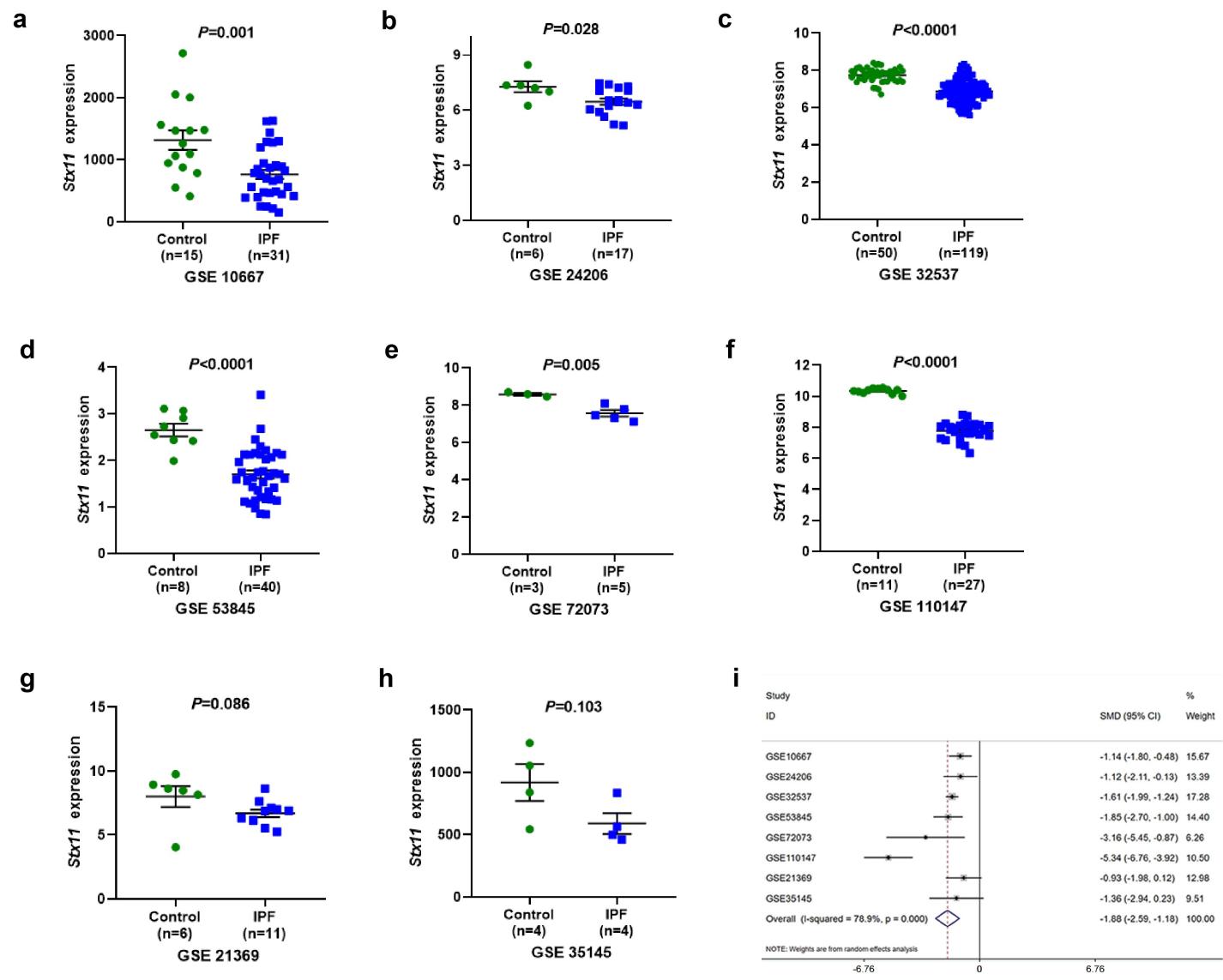


Figure. S1. The expression of STX11 in IPF and control lung tissues from the GEO database

**(a)** GSE10667, **(b)** GSE24206, **(c)** GSE32537, **(d)** GSE753845, **(e)** GSE72073, **(f)** GSE110147, **(g)** GSE21369, **(h)** GSE35145, and **(i)** meta-analysis for all data sets. CI, confidence interval; GEO, Gene Expression Omnibus; IPF, idiopathic pulmonary fibrosis. Data were expressed as mean ± SEM.


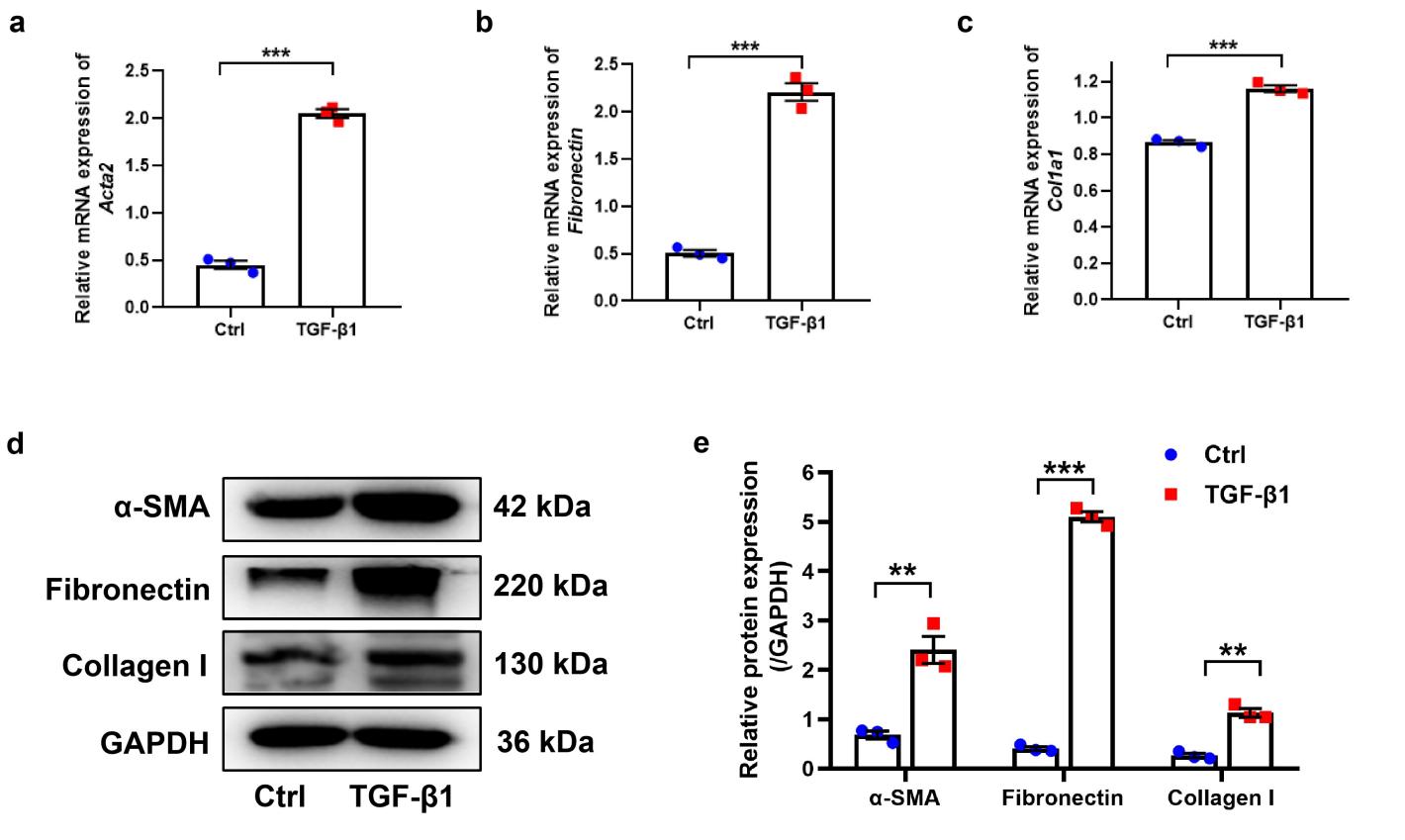


Figure. S2. The expression of fibroblast activation markers in HLFs treated with TGF-β1

HLFs were treated with 10ng/ml TGF-β1 for 48h. **(a-c)** qPCR assay was used to detect the mRNA expression of α-SMA, fibronectin, and COL1A1. **(d, e)** Western blot assay was used to detect the protein expression of α-SMA, fibronectin, and collagen I. GAPDH was used as an internal control. Data were expressed as mean ± SEM (n=3). ***p*<0.01; ****p*<0.001.


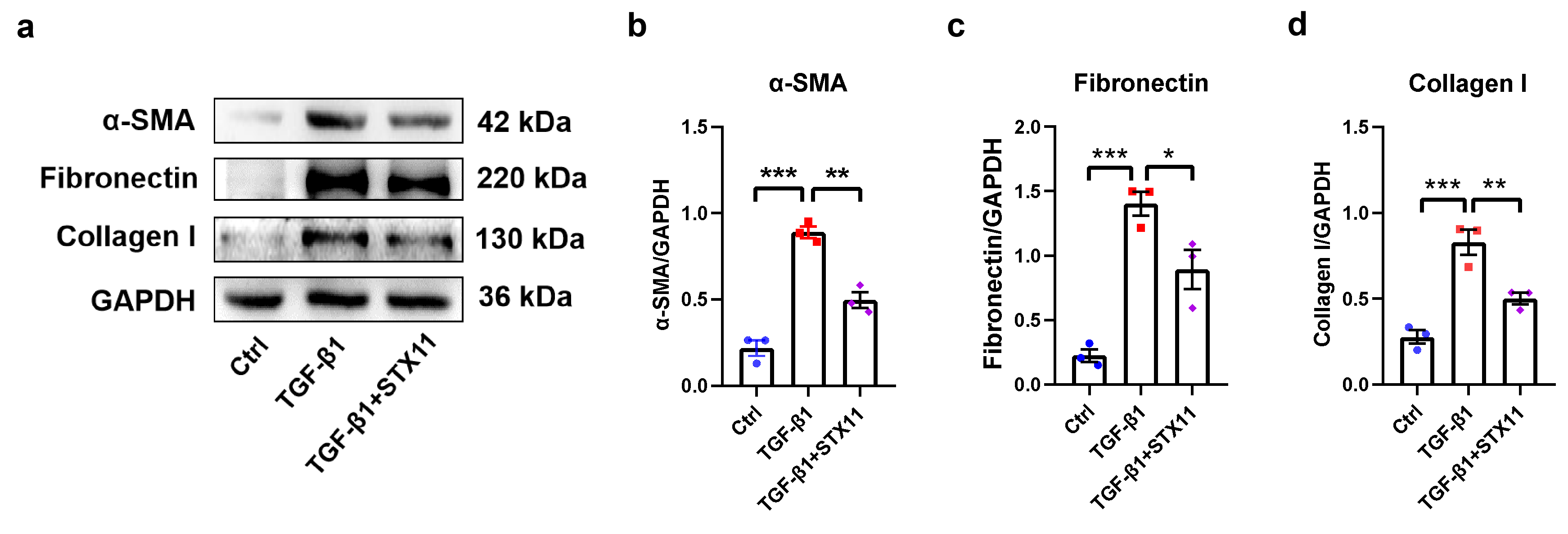


**Figure. S3. The role of STX11 in fibroblast activation**

HLFs were stimulated with recombinant human STX11 protein and TGF-β1 for 48h. **(a-d)** The protein expression of α-SMA, fibronectin, and collagen I was examined by western blot assay. GAPDH was used as an internal control. Data were expressed as mean ± SEM (n=3). *p<0.05; **p<0.01; ***p<0.001.


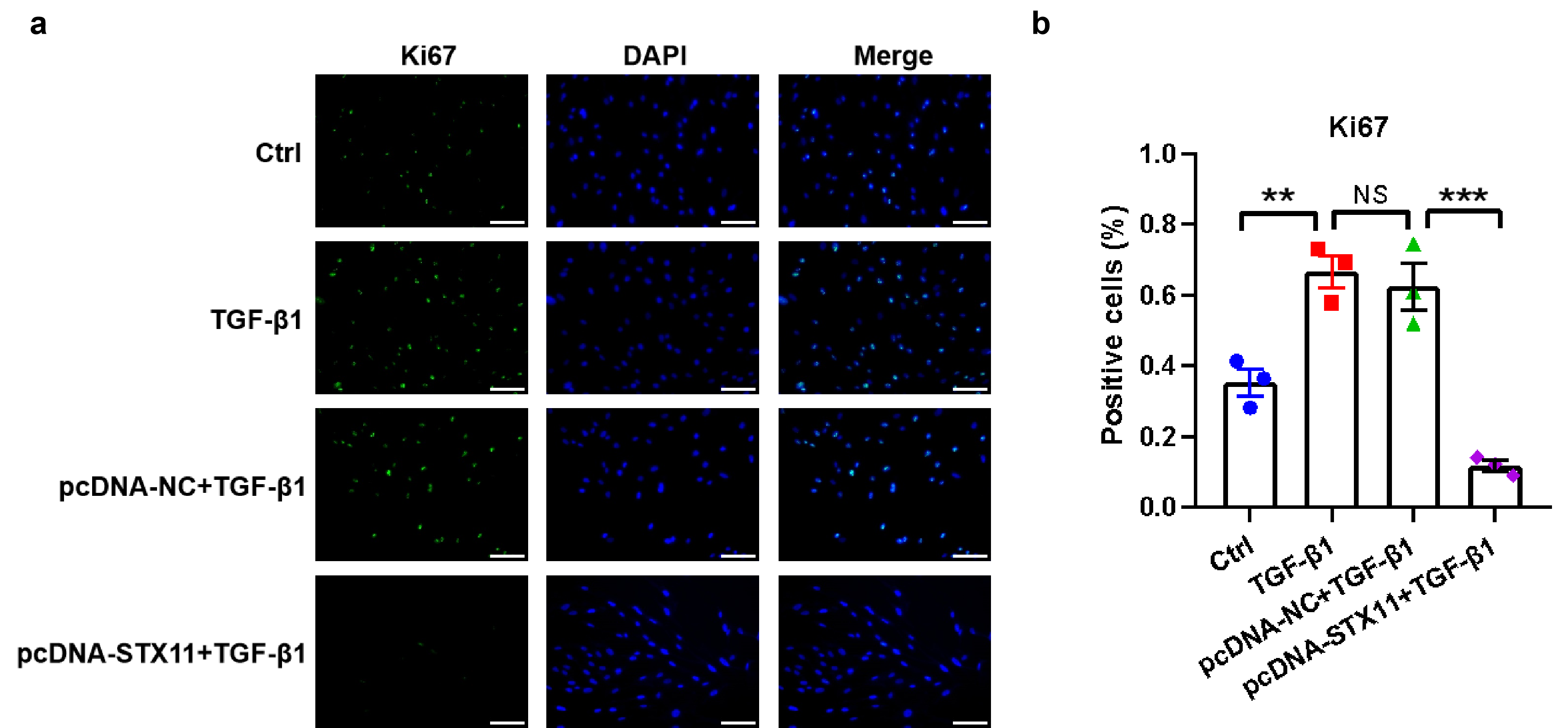


Figure. S4. The role of STX11 in fibroblast proliferation

HLFs were infected with pcDNA-STX11 plasmids for 24h, and then treated with TGF-β1 for 48h. **(a,b)** Immunofluorescence assay was used to detect Ki-67 protein expression. Scale bar=100μm. Data were expressed as mean ± SEM (n=3). ***p*<0.01; ****p*<0.001.


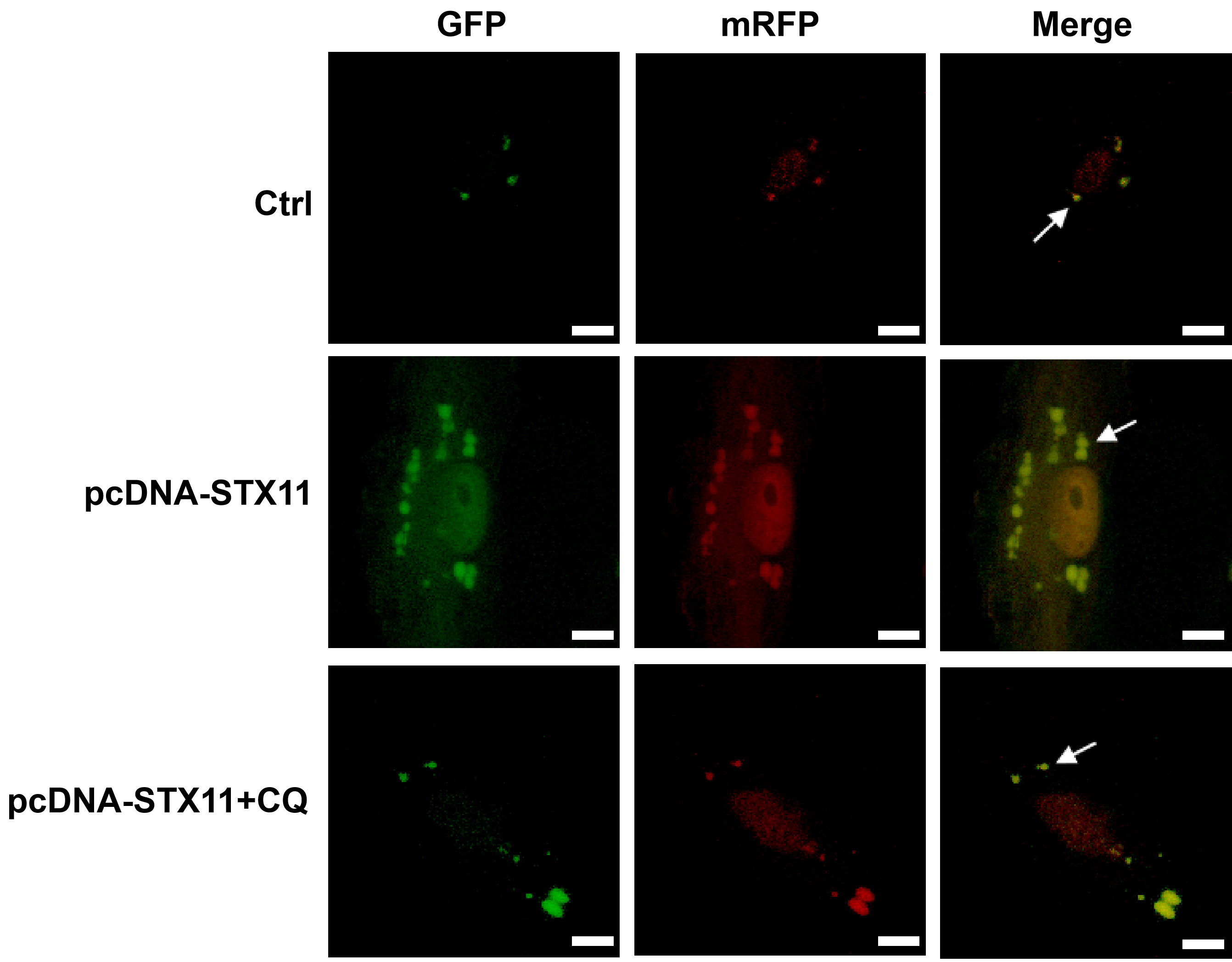


**Figure. S5. The role of STX11 in fibroblast autophagy**

The formation of autophagosomes was measured by mRFP-GFP-LC3 adenovirus infection in HLFs after overexpression of STX11 with/without stimulation of CQ for 48h (10μM). Autophagosomes were indicated by white arrows. Scale bar=10μm.


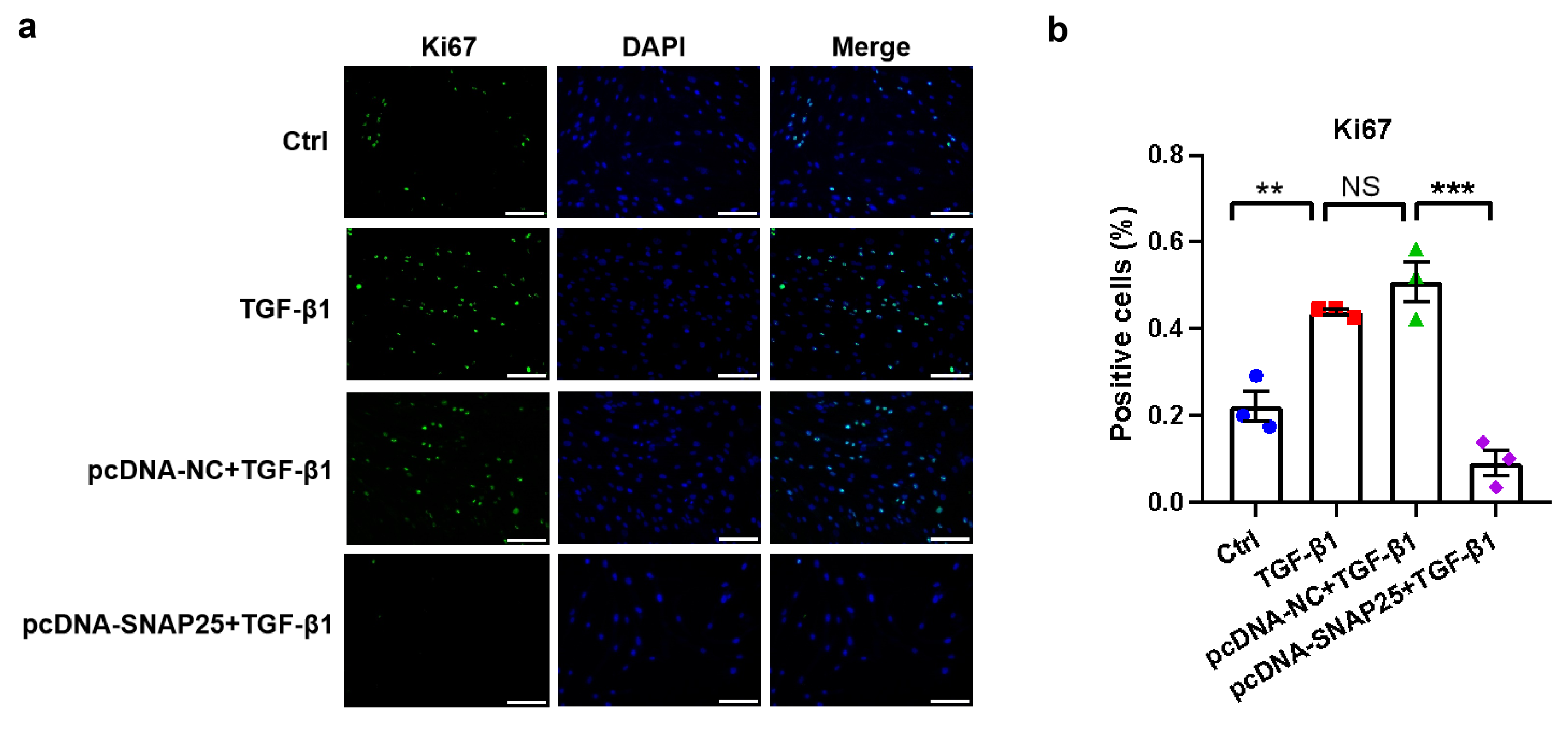


**Figure. S6. The role of SNAP25 in fibroblast proliferation**

HLFs were infected with pcDNA-SNAP25 plasmids for 24h, and then treated with TGF-β1 for 48h. **(a,b)** Immunofluorescence assay was used to detect Ki-67 protein expression. Scale bar=100μm. Data were expressed as mean ± SEM (n=3). ***p*<0.01; ****p*<0.001.


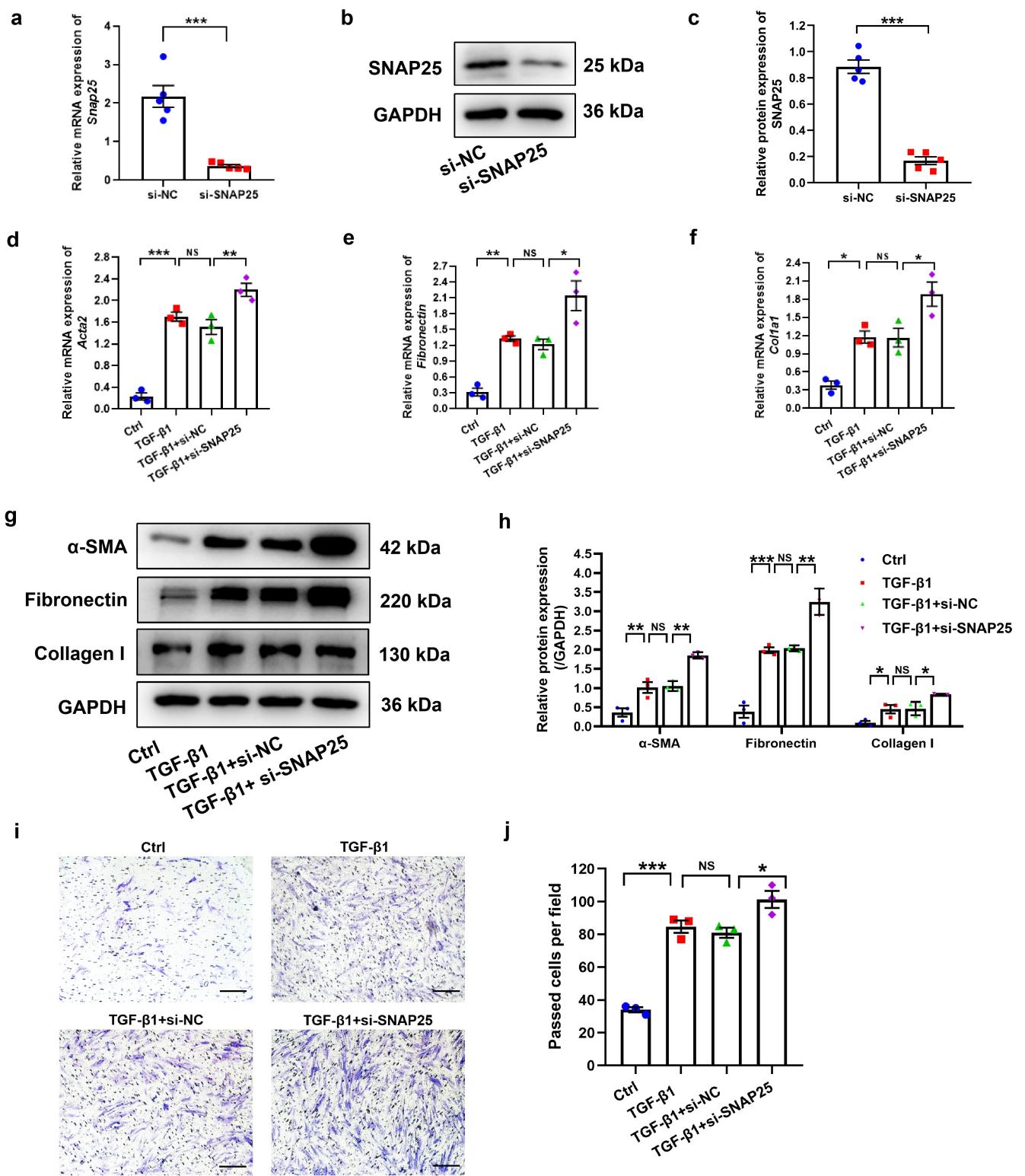


**Figure. S7. The effect of si-SNAP25 on fibroblast activation**

HLFs were transfected with si-NC or si-SNAP25 for 48h. qPCR **(a)** and western blot **(b, c)** assays were used to detect the expression of SNAP25. HLFs were transfected with si-NC or si-SNAP25 for 24, and then stimulated with TGF-β1 for 48h. qPCR **(d-f)** and western blot **(g, h)** assays were performed to determine the expression of α-SMA, fibronectin, and collagen I. **(i, j)** Transwell assay was used to determine migration of HLFs (original magnification ×100, scale bar: 200μm). GAPDH was used as an internal control. Data were expressed as mean ± SEM (n=3). **p*<0.05; ***p*<0.01 ; ****p*<0.001; NS, no significance.


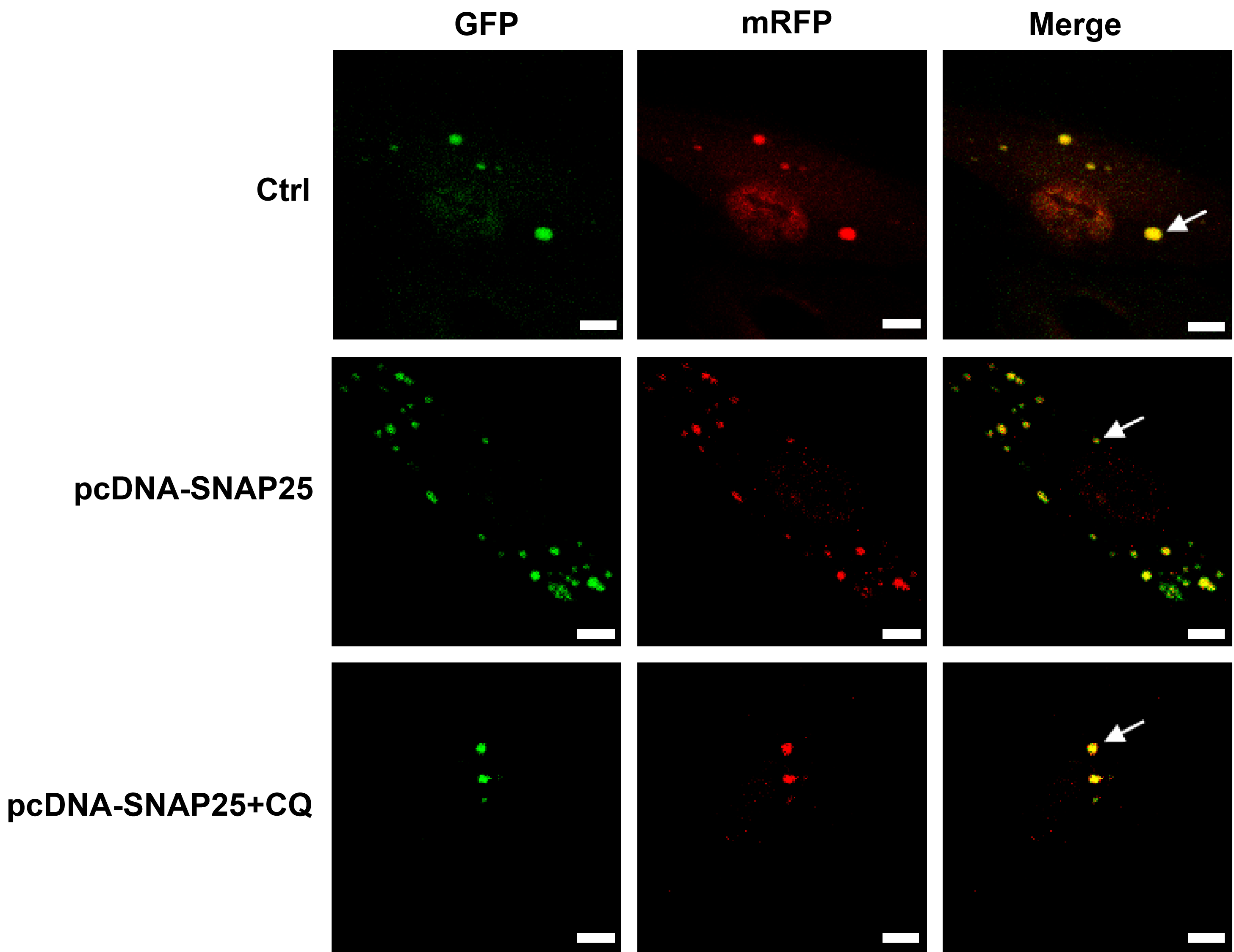


**Figure. S8. The role of SNAP25 in fibroblast autophagy**

The formation of autophagosomes was measured by mRFP-GFP-LC3 adenovirus infection in HLFs after overexpression of SNAP25 with/without stimulation of CQ for 48h (10μM). Autophagosomes were indicated by white arrows. Scale bar=10μm.
